# Validation of parent-reported physical activity and sedentary time by accelerometry in young children

**DOI:** 10.1186/s13104-015-1648-0

**Published:** 2015-11-30

**Authors:** Hrishov Sarker, Laura N. Anderson, Cornelia M. Borkhoff, Kathleen Abreo, Mark S. Tremblay, Gerald Lebovic, Jonathon L. Maguire, Patricia C. Parkin, Catherine S. Birken, The TARGet Kids Collaboration

**Affiliations:** Department of Epidemiology, Biostatistics, and Occupational Health, McGill University, Montreal, QC Canada; Pediatric Outcomes Research Team (PORT), Division of Pediatric Medicine, Department of Pediatrics, The Hospital for Sick Children, Peter Gilgan Centre for Research and Learning, 686 Bay Street, Room 10-9832, M5G 0A4, Toronto, Canada; The Applied Health Research Centre of the Li Ka Shing Knowledge Institute of St. Michael’s Hospital, University of Toronto, Toronto, ON Canada; Institute for Health Policy, Management and Evaluation, University of Toronto, Toronto, ON Canada; Children’s Hospital of Eastern Ontario Research Institute, Ottawa, ON Canada; Department of Pediatrics, Faculty of Medicine, University of Ottawa, Ottawa, ON Canada; Department of Pediatrics, St. Michael’s Hospital, Toronto, ON Canada; Department of Pediatrics, Faculty of Medicine, University of Toronto, Toronto, ON Canada

**Keywords:** Exercise, Validation studies, Child, Preschool, Sedentary lifestyle, Screen time

## Abstract

**Background:**

It is unknown if young children’s parent-reported physical activity and sedentary time are correlated with direct measures. The study objectives were to compare parent-reported physical and sedentary activity versus directly measured accelerometer data in early childhood.

**Methods:**

From 2013 to 2014, 117 healthy children less than 6 years of age were recruited to wear Actical accelerometers for 7 days. Accelerometer data and questionnaires were available on 87 children (74 %). Average daily physical activity was defined as the sum of activity ≥100 counts per minute, and sedentary time as the sum of activity <100 counts per minute during waking hours. Parents reported daily physical activity (unstructured free play in and out of school, and organized activities) and selected sedentary behaviors (screen time, stroller time, time in motor vehicle). Spearman correlation coefficients and Bland–Altman plots were used to assess the validity of parent-reported measures compared to accelerometer data.

**Results:**

Total physical activity was significantly greater when measured by accelerometer than parent-report; the median difference was 131 min/day (p < 0.001). Parent-reported child physical activity was weak to moderately correlated with directly measured total physical activity (r = 0.39, 95 % CI 0.19, 0.56). The correlations between types of physical activity (unstructured free play in and outside of school/daycare, and organized structured activity) and accelerometer were r = 0.30 (95 % CI 0.09, 0.49); r = 0.42 (95 % CI 0.23, 0.58); r = 0.26 (95 % CI 0.05, 0.46), respectively. There was no correlation between parent-reported and accelerometer-measured total sedentary time in children (r = 0.10, 95 % CI −0.12, 0.33). When the results were stratified by age group (<18, 18–47, and 48–70 months of age) no statistically significant correlations were observed and some inverse associations were observed.

**Conclusions:**

The correlation between parent-report of young children’s physical activity and accelerometer-measured activity was weak to moderate depending on type of activity and age group. Parent-report of children’s sedentary time was not correlated with accelerometer-measured sedentary time. Additional validation studies are needed to determine if parent-reported measures of physical activity and sedentary time are valid among children less than 6 years of age and across these young age groups.

**Electronic supplementary material:**

The online version of this article (doi:10.1186/s13104-015-1648-0) contains supplementary material, which is available to authorized users.

## Background

Decreased physical activity has been associated with the increased prevalence of childhood overweight and obesity in Canada [1], the United States and across Europe [2]. Physical activity is especially important during the early years of childhood, as it has been associated with improved cognition [3], mental health [4], physical health [5], and social development [6]. It is generally recommended that infants be active throughout the day through interactive floor-based play, while toddlers and preschoolers obtain at least 180 min of activity at any intensity throughout the day [7, 8]. Although there is data in the United States and Canada demonstrating low rates of physical activity in children over 6 years of age [9, 10], it is unknown if preschool children meet these guidelines. Similarly, sedentary behavior is also related to important child health outcomes [11]. While young children are not recommended to engage in sedentary behaviors (e.g., sitting in a stroller or high chair) for more than an hour at a time [12, 13], recent findings show that only 18 % of children of 3–4 years of age in Canada meet these guidelines [10]. In the United States, children less than 6 years of age spend a daily average of 2 h per day watching television [13].

In population-based studies, the most common method of studying physical activity and sedentary activity in young children is through parent-reported questionnaires [14]. Examining these types of activities and their associations with health outcomes at the population level requires valid parent-reported measures; however, only a select few studies have evaluated them in young children [15–17]. A common objective measure used to validate parent-reported physical activity questionnaires is accelerometry, which records time-stamped movement intensity in user-defined epochs [14]. A systematic review of physical activity validation studies in pediatric populations (<19 years of age) found substantial variation in studies, with low to moderate correlations with accelerometry, and only one of the 59 studies reviewed focused on children less than 5 years of age [18]. Furthermore, to the best of our knowledge, there is a lack of literature that validates the use of questionnaires in measuring sedentary time in young children, especially in those under 3 years of age. To address the important need for a feasible and low cost measure of physical activity and sedentary time in young children, the objective of this study was to validate parent-reported physical activity and sedentary time using accelerometry in children less than 6 years of age.

## Methods

### Participants

Between January 2013 and April 2014, 117 children under 6 years of age were recruited to our accelerometry study during routinely scheduled child health care visits as part of The Applied Research Group for Kids (TARGet Kids!), a primary-care, practice-based research network for children in Toronto, Canada [19]. Children were excluded from TARGet Kids! if they had health conditions affecting growth (e.g., cystic fibrosis), other chronic condition(s) (except asthma), severe developmental delay, or if their families were not able to complete questionnaires in English.

Parents were asked to attach an Actical accelerometer (Phillips—Respironics, Oregon, USA) on the right hip of their child with a velcro belt provided to them, to be worn 24 h per day for seven consecutive days, including through the night. Accelerometers were removed during bathing or swimming as they were not waterproof. Parents were provided prepaid envelopes to return the accelerometers back to the TARGet Kids!-affiliated pediatric clinics after the 7-day period.

### Parent-reported physical activity

Parents completed questionnaires, which included measures of physical activity and sedentary behaviors, based on the Canadian Health Measures Survey [20]. Parents were asked “On a typical *weekday*, how much time does your child spend outside or in a gymnasium for ‘recess’ or ‘unstructured free play’: (a) during child care/school; (b) during preschool program/daycare; and (c) aside from child care and preschool program/school and daycare?” Parents were also asked the following question about structured physical activity: “On a typical *weekday* how much time does your child spend in organized physical activities (ex. swimming, soccer, gymnastics, etc.)?” This question was also repeated asking about a typical *weekend day*. Total daily physical activity was defined as the sum of the three unstructured free play responses and the weighted average of weekday and weekend time (to better estimate the time on an average day) spent engaging in structured physical activity. Details of how physical activity was derived from the parent-report questions and accelerometry are presented in Additional file 1: Table S1.

### Parent-reported sedentary time

Parents recorded how often their children engaged in selected sedentary behaviors, as follows: “On a typical *weekday* how many minutes did your child spend awake in a room with: (a) the television on; (b) videos or a DVD on; (c) playing the computer; (d) playing video game consoles (e.g., Playstation, Xbox, Nintendo Wii); (e) playing handheld devices (e.g., iPhones, iPads, tablets, Nintendo DS video games)?” This question was asked for a typical *weekend day* as well. Parents were also asked about stroller time (“On a typical weekday, how much time does your child spend in a stroller?”) and motor vehicle time (“On a typical weekday, how much time does your child spend as a passenger in a motor vehicle (e.g., a car, bus)?”). Daily sedentary time was defined as the sum of the weighted average of total screen time, stroller time, and motor vehicle time. Details of how sedentary time was derived from the parent-report questions and accelerometry are presented in Additional file 2: Table S2.

### Accelerometer data reduction

Children with at least four valid days of accelerometer wear-time were included in the analysis [21]. These valid days could have been any combination of both weekdays and weekend days, as we have shown previously that any 2 days of accelerometer monitoring can be used to assess usual physical activity in children under 5 years of age [22]. A valid day was defined a priori as a minimum of 5 h of wear-time between 8:00 am and 8:00 pm [23]; however, there were no children in our study with less than 7.86 h wear time within the specified 12 h period. Accelerometer data were analyzed in counts per minute. The following cut-points were used to define physical activity intensity [24, 25]: sedentary time = less than 100 counts per minute (cpm); light physical activity = 100–1149 cpm; MVPA = 1150 or more cpm. Total physical activity (light and MVPA) was derived by summing all minutes equal to or greater than 100 cpm across each valid day, averaging over valid days used for each child. Similarly, daily sedentary time was derived by summing all minutes with less than 100 cpm for each valid day and calculating the average over the respective number of valid days.

### Ethics

Ethics approval to carry out the study was obtained from the Hospital for Sick Children Research Ethics Board. Written informed consent was obtained from parents. Participation was voluntary; at any given time, children could opt out from wearing the accelerometers and parents could opt out from completing the questionnaire.

### Statistical analysis

Parent-reported questionnaire data on both physical activity and sedentary time were not normally distributed and thus non-parametric tests were used. Descriptive statistics were calculated to describe characteristics of the study population. The median differences between parent-reported and accelerometer-measured physical activity and sedentary time were calculated and statistical significance was evaluated using the Wilcoxon signed-rank test. Validity of the parent-reported questionnaire measures of physical activity and sedentary time was measured by evaluating the correlation with accelerometer data using Spearman’s rank correlation. Correlation coefficients were calculated between accelerometer data and each parent-reported physical activity and sedentary behavior, as well as the total activity. Statistical significance was defined as p < 0.05, all tests were two-sided, and confidence intervals were determined using bootstrapping [26, 27]. Bland–Altman plots with mean differences and their confidence intervals were created to assess the agreement between accelerometer data and parent reported data. All statistical analyses were performed using R version 3.0.1 (R Core Team, Vienna, Austria).

## Results

**Fig. 1:**
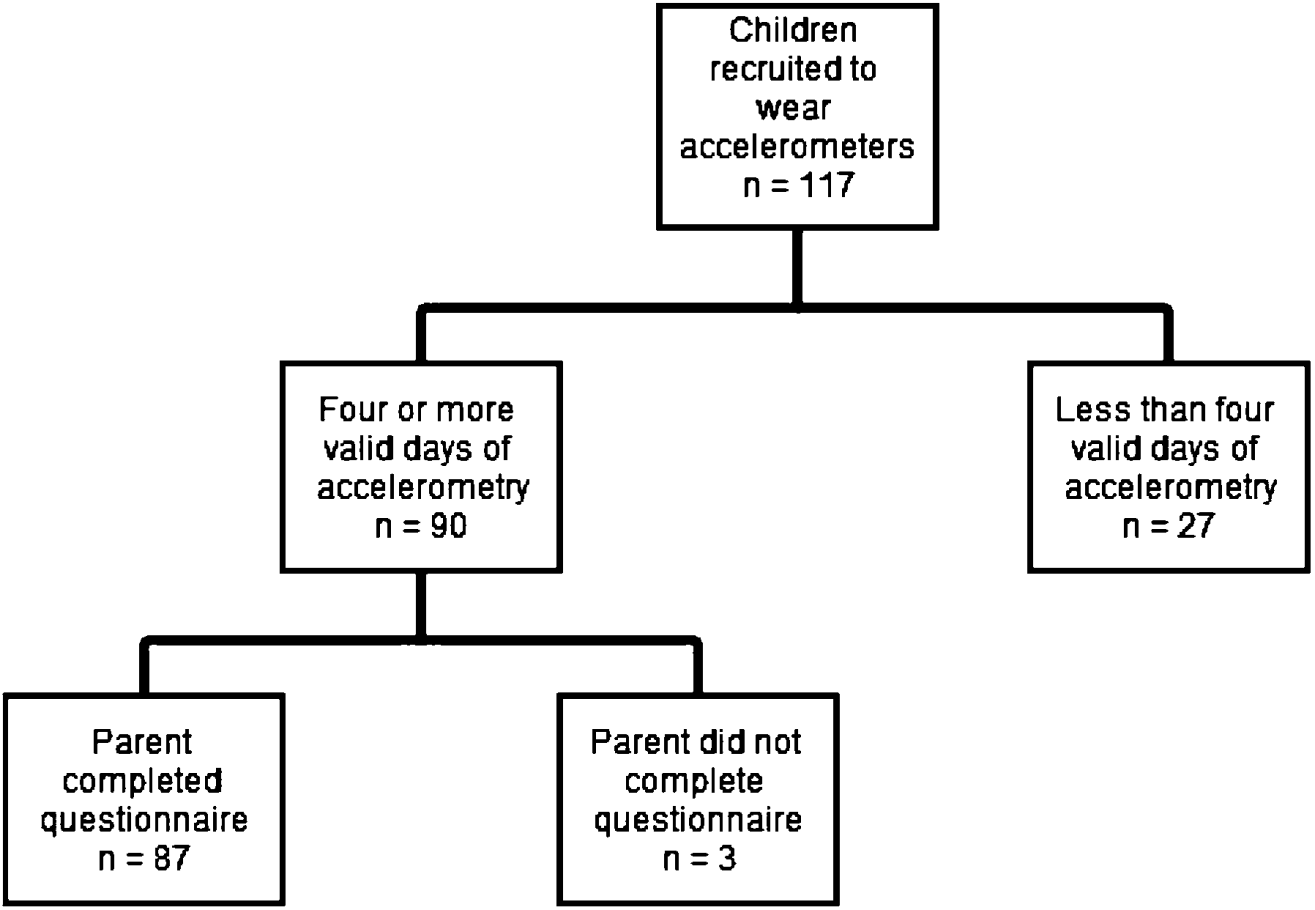
Study flow diagram

A total of 117 children were recruited to wear the accelerometers (Fig. 1). The number of children with at least four valid days of data was 90 (77 %). Three participants had missing data on physical or sedentary activity questionnaires, and thus the final sample was 87 (74 %). The age of the 87 children ranged from 4 to 70 months. Almost half (48 %) of the children were between 18 and 59 months of age and 54 % were females (Table 1). Of the sample, 77 % of children came from households with reported income greater than $100,000, and 61 % had mothers of European descent. The average number of days that children wore the accelerometer was 6.07 ± 0.74 and the average daily wear time was 10.25 ± 1.17 h (between 8:00 am and 8:00 pm).

**Table 1:** Descriptive characteristics of children with a minimum of 4 days accelerometer data (n = 87)

Characteristic	n (%)
Age (months)	
4–17	28 (32.2)
18–47	27 (31.0)
48–70	32 (36.7)
Sex	
Females	47 (54.0)
Males	40 (46.0)
Gross household income	
$150,000 or more	41 (47.1)
$100,000–$149,999	26 (29.9)
$60,000–$99,999	12 (13.8)
<$60,000	6 (6.9)
Missing	2 (2.3)
BMI z-score (based on WHO)	
<1.0 (normal weight)	72 (82.8)
1.0–2.0 (overweight)	13 (14.9)
>2.0 (obese)	2 (2.3)
Maternal ethnicity	
European	53 (61.0)
Asian	17 (19.5)
Other	11 (12.6)
Missing	6 (6.9)
Valid days of accelerometry, mean ± SD	6.07 ± 0.74
Wear time per day in hours*, mean ± SD	10.25 ± 1.17

* Wear time between 8:00 am and 8:00 pm

**Fig. 2:**
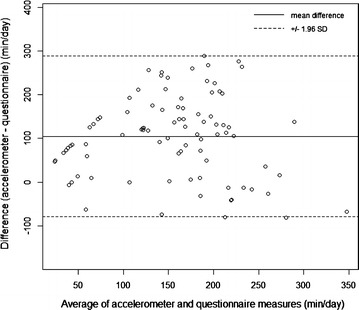
Bland–Altman plot of accelerometer- and questionnaire-measured total child physical activity

Total physical activity was significantly greater when measured by accelerometer than parent-report; the median difference was 131 min/day (p < 0.001) (Table 2). Parent-reported child total physical activity was significantly correlated with the direct accelerometer-measured physical activity (ρ = 0.39; 95 % CI 0.19, 0.56). The three individual components of parent-reported child total physical activity: free play outside a school/daycare setting; free play inside a school/daycare setting; and structured physical activity were also each significantly correlated with accelerometry (Table 3) when children of all ages were combined. The Bland–Altman plot for total physical activity is shown in Fig. 2 and suggests relatively constant variance around the mean difference. It shows a consistent discrepancy of approximately 100 min/day between accelerometer-measured and parent-reported total physical activity.

**Table 2:** Absolute differences between median parent-reported and accelerometer-measured child physical activity and sedentary time

	Parent-reported			Accelerometer-measured				
	Median	Min	Max	Median	Min	Max	Difference in median	p value*
Total physical activity (min/day)	90	0	381	221	27	366	131	<0.001
Daily sedentary time (min/day)	99	0	429	406	293	554	306	<0.001

* *p* value from Wilcoxon signed-rank test

**Table 3:** Correlation analysis between parent-reported and accelerometer-measured child physical activity and sedentary time

Variable_parent-report for a typical day_	Spearman correlation (rho)	95 % CI	p value
Total physical activity	0.39	(0.19, 0.56)	<0.001
Outdoor unstructured free play aside from school/daycare setting	0.30	(0.09, 0.49)	0.005
Unstructured free play in school/daycare setting	0.42	(0.23, 0.58)	<0.001
Structured physical activity (e.g., sports)	0.26	(0.05, 0.46)	0.015
Daily sedentary behavior	0.10	(−0.12, 0.33)	0.337
Screen time	−0.05	(−0.27, 0.18)	0.648
Stroller time	0.31	(0.09, 0.50)	0.004
Motor vehicle time	−0.09	(−0.30, 0.13)	0.412

**Fig. 3:**
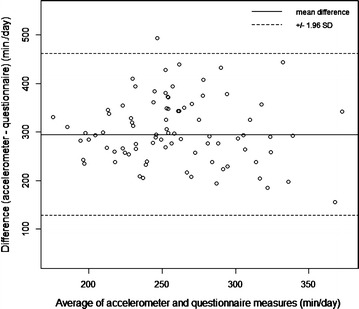
Bland–Altman plot of accelerometer- and questionnaire-measured daily child sedentary behavior

Daily sedentary time measured by accelerometry was also significantly greater than parent-report; the median difference was 306 min/day (p < 0.001) (Table 2). Parent-reported stroller time was the only sedentary activity significantly correlated with accelerometry (ρ = 0.31; 95 % CI 0.09, 0.50). Parent-reported and accelerometer-measured daily child sedentary time were not significantly correlated (ρ = 0.10; 95 % CI −0.12, 0.33), nor were daily screen time or motor vehicle time (Table 3). The Bland–Altman plot for daily sedentary activity displayed constant variance around the mean difference (Fig. 3).

**Fig. 4:**
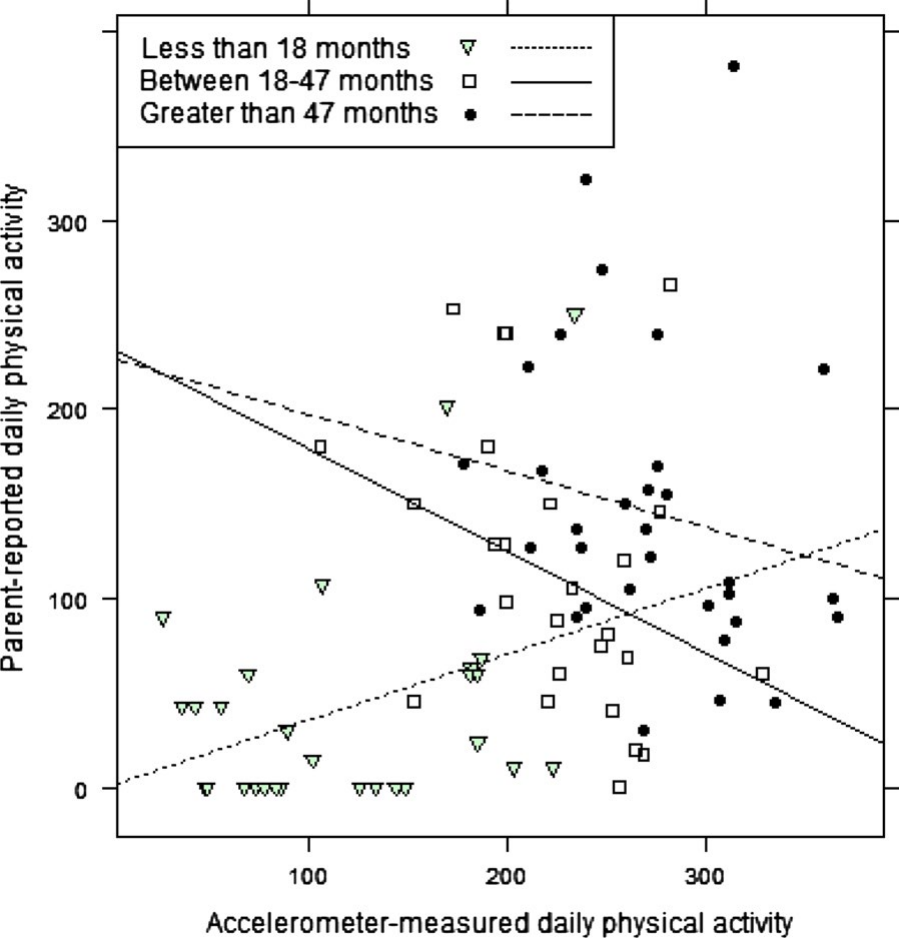
Correlations between accelerometer-measured and parent-reported total child physical activity by age group

Sensitivity analysis was conducted evaluating the validity of parent-reported and accelerometer measured physical activity using moderate- to vigorous- physical activity (MVPA) only. Parent-reported child total physical activity was compared to accelerometer-measured MVPA and the correlation between measures remained statistically significant (ρ = 0.40; 95 % CI 0.21, 0.56). In exploratory post hoc analysis, we evaluated the associations between parent-reported child total physical activity with accelerometer-measured total physical activity stratified by age groups: <18, 18–47 and 48–70 months of age (Fig. 4), as these groups reflect developmental milestones (e.g. independent ambulation at 18 months, starting school at 4 years). None of the correlations were statistically significant and wide confidence intervals were observed in all of the stratified analysis: in children <18 months ρ = 0.25 (95 % CI −0.16 to 0.60); in children 18–47 months ρ = −0.37 (95 % CI −0.73, 0.07); and in children 48–70 months of age ρ = −0.29 (95 % CI −0.60, 0.08).

## Discussion

This study provides early evidence regarding the validity of parent-reported physical activity for young children. Among all children less than 6 years of age, weak-to-moderate correlations were observed between young children’s parent-reported physical activity and accelerometer-measured total physical activity, suggesting that parent-report may be a valid measure of physical activity in early childhood. However, when our results were stratified by age, the positive correlation did not persist for all age groups and some inverse associations were observed. Although parent-reported measures of unstructured physical activity (both inside and outside daycare or school setting) and total physical activity were moderately correlated with accelerometer-measured total physical activity when all age groups were combined, these associations did not persist when stratified by age. While it is surprising that the correlation between parent-reported daycare/school physical activity and accelerometer-measured total physical activity was the highest, daycares and schools generally follow consistent daily schedules that promote activity, which may help parents measure their children’s activity. Further, parent-reported child structured activity was weakly correlated with accelerometer-measured total physical activity and its inclusion as a questionnaire item did not substantially improve the validation correlation. This may be expected for children less than 6 years of age, as most of a young child’s daily physical activity is unstructured [28].

In terms of absolute measurement, parents under-reported their child’s total physical activity by about 2 h per day (median difference of 131 min/day). A previous review of validation studies of physical activity in children found that parent-reported measures usually overestimate physical activity as compared to direct measures; however, most previous validation studies have been conducted in older children [18]. It is possible that parents of younger children may have interpreted “physical activity” as referring to high intensity activities (e.g., jumping, walking, running, etc.) and therefore generally under-reported physical activity. Total physical activity may not have been adequately captured by our questionnaire (e.g., unstructured physical activity on weekends was not captured). It is also possible that accelerometer measures in very young children may reflect other movements, such as being carried or pushed in a stroller [29].

Parent-reported daily sedentary activity was weakly correlated with accelerometer-measured daily sedentary time, suggesting that children’s daily sedentary activities were not adequately captured by our limited questionnaire items. For example, daytime naps are common in early childhood and can range from 60 to 180 min [30] and were not measured. Other activities in this age group, such as being read to, quiet activities (e.g., arts and crafts), playing with toys, and sitting to eat were also not included [31]. Parents under-reported daily sedentary activity by about 5 h per day (median difference of 306 min/day). Parents may have under-reported sedentary activities such as watching television due to social desirability [32].

The magnitude of our correlation coefficients falls between those previously found in the literature of children less than 12 years of age [15–17]. One previous study that compared parent-reported child unstructured outdoor free play with accelerometry reported a correlation of 0.20 among 250 preschool children aged 2–4 [16]. A small study of 35 children aged 3–5 years with detailed measures of various daily activities (including sports, bicycling, playing, and running around), categorized based on intensity, reported a moderate correlation of 0.49 between accelerometry-measured and parent-reported MVPA [15]. Although we could not examine activities based on intensity, our correlation coefficient remained the same when parent-reported total child physical activity was compared to accelerometer-based MVPA (ρ = 0.40), versus when compared to accelerometer-measured total physical activity (ρ = 0.39).

Our findings for all ages combined demonstrate slightly higher correlations than what has been observed previously in older pediatric populations, although there was wide variation, and different parent-reported measures [18]. It is possible that higher correlations among young children may be due to parents’ increased time spent with children in this age group, allowing for better recall of their child’s activity patterns. It is important to emphasize that in our exploratory analysis stratified by age group (<18, 18–47 and 48–70 months), none of the correlations were statistically significant and the strength of the correlations varied widely by age group with inverse correlations in the older age groups. Only the correlation for children less than 18 months of age remained positive, suggesting parent-reported child physical activity is valid only in the youngest children, although this was not statistically significant. It is difficult to draw any conclusions from this stratified analysis due to the small sample sizes of only 28, 27 and 32 children for the age groups, respectively. It is possible that the positive correlation that we observed overall is biased by age as the results within each age group do not appear to be in the same direction as the overall correlation; however, with our small sample size we may not have enough power to draw any conclusions by age group.

Few studies have evaluated indirect measures of sedentary activity in children. Colley et al. validated parent-reported sedentary activity with accelerometry in children 6–11 years of age and found a weak correlation of 0.17 [17], which was similar to our correlation for daily sedentary time (ρ = 0.10; 95 % CI −0.12, 0.33). In addition to screen time, we also measured two other potential types of sedentary activities including daily stroller time and time as a passenger in a motor vehicle. However, even when we included these activities in the analysis, the correlation remained weak. Bacardi-Gascon et al. [15] included nap time (in addition to screen time) in their validation of parent-reported child sedentary activity in children aged 3–5 and found a higher correlation of 0.35 [30].

Strengths of our study included prospective collection of a wide variety of physical activity behaviors, such as free play both outside and inside a school/daycare setting, as well as structured physical activity. We collected both measures of physical and sedentary activity on each child within a short period of time, reducing inconsistencies in time between parent-report and accelerometry. The questionnaires were completed by parents immediately prior to their children wearing the accelerometer, which may have limited bias in parents’ recall. However, it may also not accurately reflect the same time period from which accelerometers recorded data. Further, both parents and children were blinded to the directly recorded data as the accelerometers had no output display. It is unlikely that young children would have reacted to the accelerometers by engaging in more physical activity [33], although it is unknown if the parents would have encouraged it during this period. Two comprehensive reviews of the physical activity validation literature in both adults [34] and pediatrics [18] identified several limitations of validation studies, one of which is assessing correlation only. While measuring the strength of the relationship between the two measures using correlation, we also evaluated the level of agreement between them using Bland–Altman plots.

A limitation of our study was the relatively small sample of 87 children, which limited our power to evaluate any differences by age group; physical activity in children less than 18 months of age is likely different than children 18–60 months and older. Post-hoc power calculations suggest that we had 85 % power to detect a Spearman correlation of at least 0.20 for the overall association (not stratified by age), assuming a confidence interval width of at least 0.30 [35]. While there is evidence of older children being less active during weekends [36], we did not collect data on weekend unstructured physical activity. Appropriate cut-points or epoch lengths for accelerometers in this young age group remains an active area of research [23]. Further, the generalizability of our findings may be limited since our study population was of relatively high socioeconomic status and of normal weight.

## Conclusions

This study provides limited evidence that parent-reported child physical activity may be valid for the overall measurement of total physical activity in young children, although it may not be valid in all age groups of young children. Our exploratory analysis by age group suggests that parent-reported physical activity and accelerometer-measured physical activity may be inversely correlated in children age 18–47 and 48–70 months, and if true, could profoundly impact the interpretability of population-based physical activity research in young children. Future studies with larger sample sizes are needed to evaluate if these inverse correlations are significant. More research is needed to evaluate if sedentary time can be accurately captured through parent-reported questionnaires for children less than 6 years of age. Given the wide gap in the literature on both physical activity and sedentary time in young children, future studies with larger sample size are needed to evaluate the validity of parent-reported physical activity and sedentary time by age group.

## Additional files

10.1186/s13104-015-1648-0 Questionnaire and accelerometer variable definitions for total physical activity.
10.1186/s13104-015-1648-0 Questionnaire and accelerometer variable definitions for sedentary behavior.

## Abbreviations

MVPA: moderate to vigorous physical activity
CPM: counts per minute
