# Supplementary material for: Validation of parent-reported physical activity and sedentary time by accelerometry in young children
Source: BMC Res Notes. 2015 Nov 30;8:735. doi: 10.1186/s13104-015-1648-0 (PMC4666154; doi:10.1186/s13104-015-1648-0)
Supplement: Supplementary file 1 — 10.1186/s13104-015-1648-0 Questionnaire and accelerometer variable definitions for total physical activity. [file 13104_2015_1648_MOESM1_ESM.docx]

**Additional file Table S1: Questionnaire and accelerometer variable definitions for total physical activity**

| **Questionnaire** | | **Directly measured** |
| --- | --- | --- |
| **Questions asked to parent** | **Derived variable** | **Accelerometry (8 am – 8 pm)** |
| On a TYPICAL WEEKDAY, how much time does your child spend outside or in a gymnasium for 'recess' or 'unstructured free play':  ...during child care (ages 0-3)/ school (ages 4-5) ...during preschool program (ages 0-3)/ daycare (ages 4-5)  ...aside from child care and preschool program (ages 0-3)/ school and daycare (ages 4-5) | **free play** = sum of minutes/day for 3 unstructured free play questions (based on age group) | Cut-point of 100 counts per minute.  All minutes above this cut-point will be summed across each valid day and then averaged for each child on valid days. |
| On a TYPICAL WEEKDAY, how much time does your child spend in organized physical activities (ex. swimming, soccer, gymnastics, etc.)?  On a TYPICAL WEEKEND DAY, how much time does your child spend in organized physical activities (ex. swimming, soccer, gymnastics, etc.)? | **sports** =  [5(weekday) + 2(weekend)]/7  = minutes/day |  |
|  | **TOTAL PHYSICAL ACTIVITY =  free play + sports** |  |
