# Supplementary material for: Validation of parent-reported physical activity and sedentary time by accelerometry in young children
Source: BMC Res Notes. 2015 Nov 30;8:735. doi: 10.1186/s13104-015-1648-0 (PMC4666154; doi:10.1186/s13104-015-1648-0)
Supplement: Supplementary file 2 — 10.1186/s13104-015-1648-0 Questionnaire and accelerometer variable definitions for sedentary behavior. [file 13104_2015_1648_MOESM2_ESM.docx]

**Additional file Table S2: Questionnaire and accelerometer variable definitions for sedentary behavior**

| **Questionnaire** | | **Directly measured** |
| --- | --- | --- |
| **Questions asked to parent** | **Derived variable** | **Accelerometry**  **(8 am - 8 pm)** |
| On a TYPICAL WEEKDAY [WEEKEND DAY]* how many minutes did your child spend awake in a room with: ...the television on ...videos or a DVD on  ...playing the computer ...playing video game consoles (e.g., Playstation, Xbox, Nintendo Wii) ...playing handheld devices (e.g., iPhones, iPads, Tablets, Nintendo DS videogame) | sum minutes/day for all screen activities during weekday  sum minutes/day for all screen activities during weekend day    **screen** =  [5(weekday sum) + 2(weekend sum)]/7  = minutes/day | Time spent below 100 counts per minute. All wear time minutes below this cut-point will be summed across each valid day and averaged for each child on valid days. |
| On a typical weekday, how much time does your child spend in a stroller? | **stroller** = minutes/day |  |
| On a typical weekday, how much time does your child spend as a passenger in a motor vehicle (eg. a car, bus)? | **motor vehicle** = minutes/day |  |
|  | **DAILY SEDENTARY BEHAVIOR = screen + stroller +motor vehicle** |  |
